# Lupus Anticoagulant in Gulf War Illness and Autoimmune Disorders: A Common Pathway Toward Autoimmunity

**DOI:** 10.29245/2578-3009/2021/1.1208

**Published:** 2021-02-25

**Authors:** Lisa M. James, Rachel A. Johnson, Scott M. Lewis, Adam F. Carpenter, Brian E. Engdahl, Hollis E. Krug, Apostolos P. Georgopoulos

**Affiliations:** 1 Brain Sciences Center, Department of Veterans Affairs Health Care System, Minneapolis, MN, 55417, USA; 2 Department of Neuroscience, University of Minnesota Medical School, Minneapolis, MN 55455, USA; 3 Department of Psychiatry, University of Minnesota Medical School, Minneapolis, MN 55455, USA; 4 Department of Neurology, University of Minnesota Medical School, Minneapolis, MN 55455, USA; 5 Department of Psychology, University of Minnesota Medical School, Minneapolis, MN 55455, USA; 6 Department of Rheumatology, Department of Veterans Affairs Health Care System, Minneapolis, MN, 55417, USA; 7 Department of Rheumatology, University of Minnesota Medical School, Minneapolis, MN 55455, USA

**Keywords:** Lupus anticoagulant, Gulf War Illness, Lupus, Multiple sclerosis, Rheumatoid arthritis, Sjögren’s syndrome

## Abstract

Mounting evidence suggests that autoimmune mechanisms may underlie the chronic symptoms characteristic of Gulf War Illness (GWI). The presence of antiphospholipid antibodies including Lupus Anticoagulant (LA) are often associated with autoimmune disorders. Here we evaluated and compared blood samples from veterans with GWI and veterans with other autoimmune conditions including relapsing remitting multiple sclerosis, rheumatoid arthritis, Sjögren’s syndrome, and lupus for the presence of LA using Silica Clotting Time and dilute Russell’s Viper Venom Time assays. Positive LA was identified in one-quarter of veterans with GWI; this proportion was not statistically different from the proportion of positive LA identified in patients diagnosed with the other autoimmune conditions. The present findings add to the literature implicating autoimmune mechanisms in GWI and point to the presence of prothrombotic antiphospholipid antibodies as a common contributing factor in GWI and other autoimmune disorders. Furthermore, activation of the coagulation system suggests new potential avenues for treatment for LA-positive Gulf War veterans.

## Introduction

Gulf War Illness (GWI) continues to plague one-third of veterans of the 1990–91 Persian Gulf War with chronic, debilitating symptoms affecting multiple organ systems. Symptoms range from fatigue, widespread pain, and cognitive problems to respiratory difficulties, dermatological complaints, and gastrointestinal issues^1,2^. Mounting evidence points to immune system disruptions^3–7^ and autoimmunity^8–12^ as mechanisms underlying the widespread symptoms characteristic of GWI. Evidence for autoimmunity in GWI includes brain functional patterns that are indistinguishable from those of known autoimmune disorders^8^, clinical and pathological features that are similar to other autoimmune syndromes^9,11^, and the presence of autoantibodies to neuronal and glial proteins^12^.

Although the cause of GWI is disputed, several research groups have suggested that immune system responses to foreign antigens such as pathogens, vaccines, and/or chemical/biological warfare agents (or prophylactics against them) may underlie GWI^4, 9–17^. One theory has proposed that the immune response to such exposures may activate the coagulation system via the cross-reaction of antibodies against cell-surface antithrombotic proteins^17^. As theorized, evidence of blood hyper-coagulation was found in veterans with GWI^17^. That study also reported evidence of antiphospholipid antibodies (i.e., anti Beta-2-glycoprotein I; aβ2GPI) in some veterans with GWI. Antiphospholipid antibodies including aβ2GPI, anticardiolipin (aCL), and lupus anticoagulant (LA) are autoantibodies against phospholipids or phospholipid-binding proteins that result in formation of blood clots in vivo. Of the antiphospholipid antibodies, LA is the strongest risk factor for prothrombotic events^18^ and has been associated with increased risk of myocardial infarction^19^. Positive LA has been reported in patients with various autoimmune conditions including lupus (26–34%^19,20^), Sjögren’s syndrome (9–11%^21,22^), rheumatoid arthritis (6.6%^23^), and multiple sclerosis (22%^24^). Here we evaluate and compare the presence of positive lupus anticoagulant in GWI and four known autoimmune disorders to further characterize GWI with regard to autoimmune processes and inform potential treatment avenues.

## Materials and Methods

A total of 148 veterans (126 men) participated in this study as paid volunteers. Participants belonged to one of the following 5 disease groups. (1) GWI without any autoimmune conditions (N = 84; 77 men); (2) relapsing remitting multiple sclerosis (RRMS; N = 18; 14 men); (3) rheumatoid arthritis (RA; N = 24; 22 men); (4) Sjögren’s syndrome (SS; N = 8; 4 men); and (5) lupus (Lupus; N = 14; 9 men). GWI patients met criteria for both Centers for Disease Control^1^ and Kansas^2^ case definitions. Diagnoses for autoimmune patient groups were established by specialists in the rheumatology or neurology clinics at the Minneapolis Veterans Affairs Health Care System. The mean ± SEM age (y) was 56.4 ± 0.90 for GWI, 45.3 ± 2.92 for RRMS, 66.5 ± 1.26 for RA, 58.0 ± 4.47 SS, and 62.3 ±2.29 Lupus. The Minneapolis VA Health Care System (VAHCS) Pathology and Laboratory Medicine Service evaluated blood samples for markers related to autoimmunity, including Lupus Anticoagulant (LA). LA was assessed using a standard Lupus Inhibitor Panel (VA Document ID:HEM02–023, issue date: 11/4/2019) consisting of Silica Clotting Time (SCT) and dilute Russell’s Viper Venom time (dRVVT), with both screen and confirm tests being run simultaneously for both tests. Cutoff values were 1.19 for SCT C test ratio and 1.15 for dRVVT C test ratio; the Lupus Inhibitor Panel outcome was Negative if both values were below cutoffs and Positive if either value was above the cutoff. Thus, for each group, the number of participants with positive LA test was available, from which the proportion of positives was calculated. This proportion of LA positive in the GWI group was compared to the corresponding proportions of the other groups using the Wald H0 test for independent-samples proportions (IBMSPSS statistical package, version 27). Anticoagulants have been associated with false-positive lupus anticoagulant tests^25^; none of the participants in the present study were taking anticoagulants.

Each participant in the GWI group completed a standard GWI symptom questionnaire with six symptom domains^2^: fatigue, pain, neurological-cognitive-mood, gastrointestinal, respiratory, and skin. Individual symptom severity was reported in a scale from 0 to 3. For each participant, an average score per domain was calculated and used for comparisons between GWI participants that were LAC-positive or LAC-negative. using an independent samples t-test.

## Results

**Figure 1:**
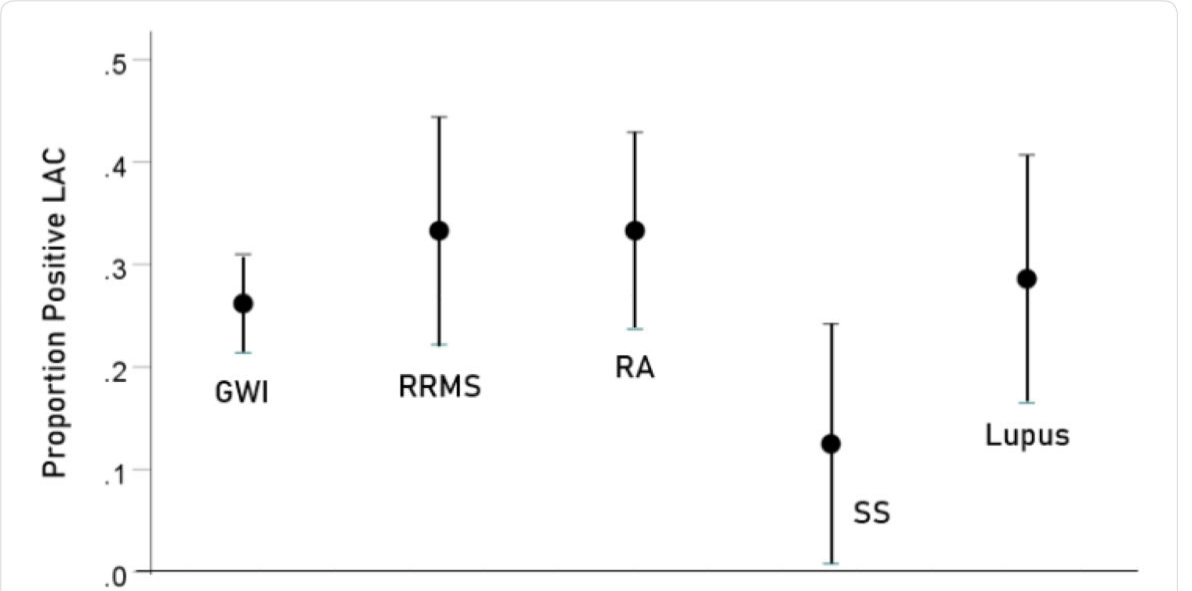
Proportion of positive LAC ± 1 standard error in each group.

Table 1 shows the proportions and associated statistics of LA observed in all disease groups (also see Figure 1). It can be seen that they were all similar, >25% for all but SS. Table 2 shows the results of testing these proportions between all disease pairs; none differed significantly from each other.

## Discussion

In the present study we evaluated blood samples for the presence of positive LA in veterans with Gulf War Illness and compared the proportion of positive LA tests to that observed in other autoimmune disorders. The findings revealed the presence of positive LA in one-quarter of veterans with GWI, a rate that was comparable to that found in veterans diagnosed with autoimmune disorders. To our knowledge, this is the first study to demonstrate the presence of LA in Gulf War Illness, complementing findings of a previous study demonstrating the presence of another antiphospholipid antibody, B2GPI, in veterans with GWI^17^. The present findings add to the literature implicating autoimmune mechanisms in GWI and specifically point to the involvement of prothrombotic antiphospholipid autoantibodies in GWI.

Several researchers have characterized GWI as an autoimmune condition^8–12^. Environmental exposures to infectious agents^26^ and vaccines^27^ have been widely implicated in the development of various autoimmune conditions, and similarly, have been implicated in GWI^9–11,14–16^. Furthermore, several infectious agents^28,29^, including, most recently, SARS-CoV-2^30^, and vaccines^29^ have been linked to the presence of antiphospholipid autoantibodies. In both infections and vaccines, phospholipid autoimmunity is purported to primarily occur as a result of molecular mimicry between phospholipid proteins and those of foreign antigens^28,29^. The extent to which Gulf War exposures (e.g., infectious agents, vaccines, chemical/biological warfare agents) may similarly share peptide sequences with phospholipids, thereby contributing to autoimmunity, remains to be investigated. In addition, chronic inflammation resulting from war-related environmental exposures, independently or coupled with stress, may result in tissue damage that, in turn, triggers autoimmunity. It is worth noting that the prevalence of lupus anticoagulant across all veteran patient groups in this study was somewhat higher here than what has previously been reported in the literature^19–24^. This raises the possibility that some environmental exposures that may contribute to autoimmunity are more common among the veteran population relative to their non-veteran counterparts.

The current finding that veterans with GWI and autoimmune disorders show comparable evidence of positive lupus anticoagulant further substantiates theories of GWI as an autoimmune condition^8–11^; however, only a subset of veterans with GWI (or other autoimmune disorders) in the current study exhibited positive LA, suggesting the influence of additional mechanisms, including potentially other immune-mediated processes. For example, GWI has been linked to lack of immunogenetic protection^4^ in which a mismatch between an individual’s human leukocyte antigen (HLA) composition and foreign antigens to which they are exposed (e.g., anthrax^14,15^) leads to persistent antigens that contribute to disease^16,31^. Of note, certain HLA alleles have also been associated with the presence of autoantibodies including lupus anticoagulant^32^. Future research evaluating the influence of HLA genotype on autoimmunity in GWI may prove informative with regard to understanding genetic susceptibility toward different manifestations of GWI.

Although the findings of the current study provide novel insights regarding autoimmune mechanisms associated with GWI, the findings must be considered within the context of study limitations. First, lupus anticoagulant was the only antiphospholipid antibody investigated in this study. With the exception of one prior study that reported positive B2GPI autoantibodies in three of the five GWI veterans that were evaluated^17^, the extent to which other antiphospholipid antibodies are present in these veterans, potentially contributing to autoimmunity, is unknown. Second, antiphospholipid antibody concentrations can fluctuate over time; however, these analyses were based on a single time point. Third, a veteran control group was not included in the present study, precluding comparison of LA-positivity in control veterans to the rates found in the present sample. Finally, this was a cross-sectional study. Longitudinal studies will be necessary to evaluate the long-term prognosis for Gulf War veterans with positive LA relative to those without positive LA. For instance, researchers have described a crescendo of autoimmunity characterized by increased concentrations of autoantibodies and increasingly varied antibodies in SLE^33^. It remains to be seen if a similar process is characteristic of LA-positive GWI.

## Conclusion

The present findings suggest that for at least a nontrivial sub-population of veterans with GWI, activation of the coagulation system is an outcome that influences GWI and overlaps with known autoimmune disorders. In light of recent findings that male Gulf War veterans have significantly greater risk of stroke and myocardial infarction compared to the general population^34^, and increased risk of deep vein thrombosis/pulmonary embolism in female Gulf War veterans^35^, the current findings highlight the potential utility of routine monitoring for evidence of hypercoagulation in veterans with GWI and interventions aimed at reducing the likelihood of thrombotic events in LA-positive GWI veterans.

## Figures and Tables

**Table 1:** Observed proportions and associated statistics of LA in the 5 disease groups.

	N of LA present	Total N	Proportion	Asymptotic Standard Error
GWI	22	84	0.262	0.048
RRMS	6	18	0.333	0.111
RA	8	24	0.333	0.096
SS	1	8	0.125	0.117
Lupus	4	14	0.286	0.121

**Table 2.** Two-sided probability values of the Wald H0 test of the difference between two proportions.

	GWI	RRMS	RA	SS	Lupus
GWI	X				
RRMS	0.538	X			
RA	0.491	1.000	X		
SS	0.393	0.269	0.256	X	
Lupus	0.852	0.773	0.761	0.387	X

With respect to GWI symptom severity, no statistically significant differences were found for any of the 6 symptom domains above (P > 0.05 for all comparisons, uncorrected for multiple comparisons).
